# Cold Tolerance during the Reproductive Phase in Chickpea (*Cicer arietinum* L.) Is Associated with Superior Cold Acclimation Ability Involving Antioxidants and Cryoprotective Solutes in Anthers and Ovules

**DOI:** 10.3390/antiox10111693

**Published:** 2021-10-26

**Authors:** Anju Rani, Asha Kiran, Kamal Dev Sharma, P. V. Vara Prasad, Uday C. Jha, Kadambot H. M. Siddique, Harsh Nayyar

**Affiliations:** 1 Department of Botany, Panjab University, Chandigarh 160014, India; thakur.aanjali@gmail.com; 2 Department of Agricultural Biotechnology, CSK Himachal Pradesh Agricultural University, Palampur 176062, India; singhashakiran@gmail.com (A.K.); kamal@hillagric.ac.in (K.D.S.); 3 Department of Agronomy, Kansas State University, Manhattan, KS 66506, USA; 4 Crop Improvement Division, Indian Institute of Pulses Research, Kanpur 208024, India; u9811981@gmail.com; 5 The UWA Institute of Agriculture, The University of Western Australia, Perth, WA 6009, Australia; kadambot.siddique@uwa.edu.au

**Keywords:** chilling, legumes, pollen, stigma, acclimatization, stress

## Abstract

Chickpea is sensitive to cold stress, especially at reproductive stage, resulting in flower and pod abortion that significantly reduces seed yield. In the present study, we evaluated (a) whether cold acclimation imparts reproductive cold tolerance in chickpea; (b) how genotypes with contrasting sensitivity respond to cold acclimation; and (c) the involvement of cryoprotective solutes and antioxidants in anthers and ovules in cold acclimation. Four chickpea genotypes with contrasting cold sensitivity (cold-tolerant: ICC 17258, ICC 16349; cold-sensitive: ICC 15567, GPF 2) were grown in an outdoor environment for 40 days in November (average maximum/minimum temperature 24.9/15.9 °C) before being subjected to cold stress (13/7 °C), with or without cold acclimation in a controlled environment of walk-in-growth chambers. The 42-d cold acclimation involved 7 d exposure at each temperature beginning with 23/15 °C, 21/13 °C, 20/12 °C, 20/10 °C, 18/8 °C, 15/8 °C (12 h/12 h day/night), prior to exposing the plants to cold stress (13/7 °C, 12 h/12 h day/night; 700 μmol m^−2^ s^−1^ light intensity; 65–70% relative humidity). Cold acclimation remarkably reduced low temperature-induced leaf damage (as membrane integrity, leaf water status, stomatal conductance, photosynthetic pigments, and chlorophyll fluorescence) under cold stress in all four genotypes. It only reduced anther and ovule damage in cold-tolerant genotypes due to improved antioxidative ability, measured as enzymatic (superoxide dismutase, catalase, ascorbate peroxidase, and glutathione reductase) and non-enzymatic (ascorbate and reduced glutathione), solutes (particularly sucrose and γ-aminobutyric acid) leading to improving reproductive function and yield traits, whereas cold-sensitive genotypes were not responsive. The study concluded that cold tolerance in chickpea appears to be related to the better ability of anthers and ovules to acclimate, involving various antioxidants and cryoprotective solutes. This information will be useful in directing efforts toward increasing cold tolerance in chickpea.

## 1. Introduction

Chickpea (*Cicer arietinum* L.), the third most important grain legume in the world, is an important source of protein to human and animals in Asia and Africa. Consequently, major chickpea growing areas lie in these two continents; however, it is also cultivated in the USA, Canada, and Australia primarily for export to Asian and African countries. Chickpea evolved in the warm climates of the Mediterranean region and is thus sensitive to low temperatures [1,2,3]. Chickpea experiences stressful low temperatures either during vegetative or reproductive growth, depending on the cultivation region [2,4,5,6]. In northern India and southern Australia, chickpea experiences low temperatures (<20/10 °C) during reproductive growth wherein cold stress damages leaves and flowers, decreases pollen and ovule fertility, impairs fertilization and alters the transcription in anthers and leaves [4], leading to flower and pod abortion and reducing the yield potential [4,6,7,8,9,10]. The threshold temperature for chickpea is 21 °C and temperatures below are stressful to chickpea; consequently, many production regions in the world are susceptible to cold stress [2,10].

Cold-stress-induced aberrations in crops at various organizational levels, including reduced vegetative and reproductive growth, delayed phenology, enhanced leaf chlorosis and necrosis, changes in leaf hydration status, flower abnormalities, and damage to reproductive structures and yield including chickpea are well understood [1,2,4,11]. Cold stress results in fewer numbers of pods and seeds per pod leading to lower yield [6]. In cold-sensitive chickpea genotypes, cold stress at all anther development stages i.e., micro- or mega-sporogenesis, gametogenesis and at mature pollen stage results in flower abortion [2]. The flower abortion is caused either by disruption of gametogenesis or abnormal pollen/ovule development that leads to sterility [2]. Younger flowers are relatively more sensitive to cold stress compared to old flowers as younger flowers do not have developed pollen grains whereas older flowers have developed pollen grains and results in sterility [2]. In older flowers, cold stress also decreases the ability of the pollen grains to germinate and retards pollen tube growth leading to failure or lack of fertilization resulting in poor seed set and fewer seeds per pod [2,4].

Despite significant advancements in our understanding of cold stress responses of chickpea, the metabolic and molecular mechanisms affecting cold sensitivity, especially in flowers, are relatively poorly understood [4,9,10]. At the cellular level, cold stress induces damage to membranes, increases production of reactive oxygen species (ROS), denatures enzymes and proteins and causes hormonal imbalance [12]. Our recent study [4] focused on the impact of cold stress on metabolites and enzymatic antioxidants as well as expression of genes of these pathways in anthers of cold-sensitive and cold-tolerant genotypes. While starch and proline were decreased in the cold-sensitive genotypes, there was no change in the cold-tolerant genotype. This decrease in sensitive genotype resulted from down-regulation of sucrose and proline transporter genes whereas there was up-regulation of these genes in cold-tolerant genotype [4]. It was shown that pollen viability of cold-tolerant genotypes was linked to maintenance of starch, reducing sugars and proline levels [4]. Additional studies are, however, needed to elucidate complete mechanisms associated with cold-induced flower abortion.

Plants, even cold-sensitive ones, also possess the ability to acquire cold tolerance. Cold tolerance acquisition takes place when plants are exposed to gradually decreasing low non-freezing temperatures, a process known as cold acclimation [13]. In general, acclimated plants may have greater cold tolerance compared to plants those are not acclimated [14,15,16]. Cold acclimation has been reported in several crops such as oilseed rape (*Brassica napus*; [17]), barley (*Hordeum vulgare*; [18]), and *Arabidopsis thaliana* [19]. In oilseed rape, maximum cold tolerance was achieved by exposure to 3 d of acclimation in spring cultivars and between 6 and 9 d in the winter cultivars, and cold tolerance decreased with prolonged acclimation duration [17]. At physiological level, the cold acclimation in barley led to significant changes in tissue water content, carbohydrate content and resulted in improved tillers and growth compared to non-acclimated plants [18]. Cold acclimation also modified the photosynthetic machinery and enabled plants to survive under severe cold temperatures through manipulation of chlorophyll a fluorescence [19]. Though information is not available for chickpea, in other crops, cold acclimation encompasses several mechanisms involving membrane changes [14], osmoprotectant accumulation (e.g., carbohydrates, proline, glycine betaine), antioxidant up-regulation [13,15] coupled with changes in expression of genes of these pathways [16]. These modifications during cold acclimation prepare cells to tolerate subsequent stressful low temperatures. It appears that the differential ability of the crops or their genotypes to tolerate cold stress depends on the types of physiological or biochemical changes during the process of cold acclimation.

The impact of cold acclimation on chickpea is not well documented or understood, although a few studies showed the benefits of cold acclimation during early vegetative growth [9,20,21]. There is no information on reproductive benefits of cold acclimation in chickpea. It was hypothesized that antioxidants (enzymatic or non-enzymatic) and solutes (e.g., osmolytes and carbohydrates) accumulate in chickpea on exposure to gradually decreasing temperatures and result in cold acclimation. It is also not known whether cold-sensitive and cold-tolerant genotypes behave similarly or differently upon cold acclimation. Therefore, the objectives of this study were to evaluate (a) whether cold acclimation imparts reproductive cold tolerance in chickpea; (b) whether genotypes with contrasting cold sensitivity respond similarly or differently to cold acclimation; and (c) the cryoprotective solutes and antioxidants are involved in cold acclimation in anthers and ovules.

## 2. Materials and Methods

### 2.1. Plant Growth Conditions and Treatments

**Figure 1:**
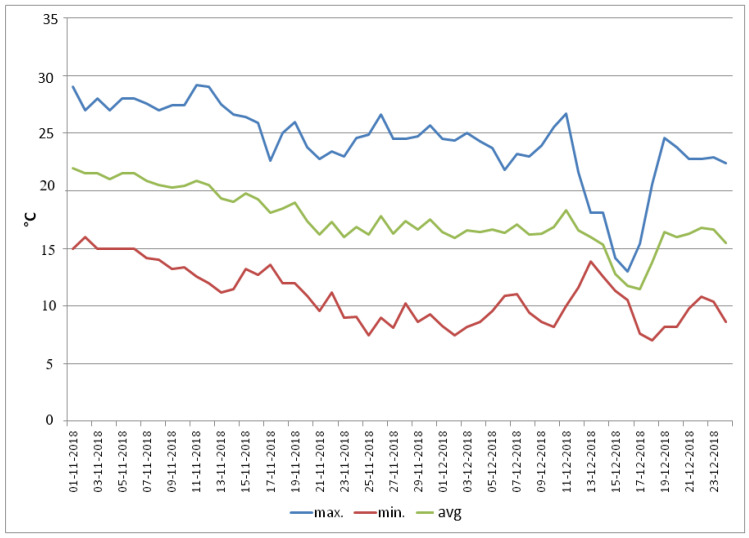
Weather data (maximum (max), minimum (min) and average (avg) temperature) from sowing up to 40 days, when the plants were moved to growth chamber.

Chickpea seeds of contrasting genotypes (cold-tolerant: ICC 17258, ICC 16349; cold-sensitive: ICC 15567, GPF 2)—selected from preliminary screening experiments involving 40 genotypes (unpublished)—were soaked for 12 h and inoculated with an appropriate culture of *Rhizobium* sp. Five inoculated seeds were sown in pots filled with sandy loam soil and farmyard manure (3:1 ratio). Tricalcium phosphate fertilizer was added (10 mg kg^–1^ soil). Fifteen days after sowing (DAS), the plants were thinned to two per pot. Sowing was undertaken in the first week of November in an outdoor natural environment in wired enclosures (to protect against birds and animals). The weather data are plotted in Figure 1 (24.9/15.9 °C mean day/night temperatures, 1300–1500 μmol m^−2^s^−1^ light intensity, 60–70% relative humidity). At 40 DAS, plants were moved into walk-in-growth chambers for the treatments:

1. Control: 25/15 °C (12 h/12 h day/night), 700 μmol m^−2^s^−1^ light intensity, and 65–70% relative humidity until maturity;
2. Non-acclimated, cold-stressed: 25/15 °C (12 h/12 h day/night), 700 μmol m^−2^s^−1^ light intensity, and 65–70% relative humidity for one day; temperature then reduced to 13/7 °C (12 h/12 h day/night) over 4 days to avoid lethal shock, where it remained at this temperature until maturity; and
3. Cold-acclimated, cold-stressed: 25/18 °C (12 h/12 h day/night), 700 μmol m^−2^s^−1^ light intensity, and 65–70% relative humidity for one day, followed by 42 d of cold acclimation, involving 7 d exposure at each decreasing temperature beginning with 23/15 °C, 21/13 °C, 20/12 °C, 20/10 °C, 18/8 °C, 15/8 °C (12 h/12 h day/night) before exposing the plants to cold stress at 13/7 °C (12 h/12 h day/night; 700 μmol m^−2^s^−1^ light intensity, and 65–70% relative humidity). Thereafter the temperature remained at 13/7 °C until maturity.

The plants were assessed for stress injury during the reproductive stages after experiencing a minimum of 10 d exposure to normal or stressful temperatures using the procedures described below. Young leaves subtending flowers were collected from the second and third nodes. Flowers were collected at the same time. Leaf traits such as stomatal conductance and photosystem II function and biochemical traits were analyzed from 3 different randomly selected young leaves subtending flowers per plant (values were averaged), in three different plants (three replications). The data were pooled, and mean values and standard errors (SE) were estimated.

### 2.2. Stress Injury

#### 2.2.1. Membrane Damage

Membrane damage was measured as electrolyte leakage (EL). Young fresh leaves located at the second/third node below flowers were collected. For analysis in anthers and ovules, flowers were collected on the day of anthesis. The tissues were washed with deionized water, dissected into smaller segments, and placed in glass vials containing 10 mL deionized water for 12 h at 25 °C. The electrical conductivity (C1) of the surrounding solution was measured after 24 h. The tissue segments were then subjected to 80 °C in a water bath for 10–15 min. The final electrical conductivity (C2) was measured after equilibration. Membrane damage was calculated as C1/C2 × 100 and expressed as a percentage [22].

#### 2.2.2. Cellular Oxidizing Ability

Cellular oxidizing ability was assessed using 2,3,5-triphenyl tetrazolium chloride (TTC) reduction ability, involving the conversion of a colorless solution into dark red formazan due to reduction by the cells. Fresh tissue (leaves, anthers, or ovules) was immersed in an incubation solution containing 50 mM sodium phosphate (pH 7.4) and TTC (500 mg 100 mL^−1^ solutions) and kept in the dark for 1 h at 25 °C, without shaking as the reduction of TTC responds to high oxygen. The tissue samples were extracted twice (5 mL each) using 95% ethanol and combined to make a final volume of 10 mL. The developed red color was measured at 530 nm using a spectrophotometer and expressed as absorbance g^–1^ fresh weight (FW) [23].

#### 2.2.3. Relative Leaf Water Content

Leaf water status was measured as relative leaf water content (RLWC). Fresh leaves weight (FW) (500 mg) were placed in Petri dishes containing distilled water for 2 h, removed, surface dried with filter paper, weighed initially (turgid weight, TW; weight of fully hydrated leaf), and weighted again after oven-dried at 110 °C for 24 h (dry weight, DW). RLWC was calculated as (FW − DW)/(TW − DW) × 100; expressed as a percentage [24].

#### 2.2.4. Stomatal Conductance

Leaf stomatal conductance was measured with a portable leaf porometer (Decagon Devices, Pullman, WA, USA) and expressed as mmol^−1^ m^–2^ s^−1^ [22].

#### 2.2.5. Photochemical Efficiency

Photochemical efficiency was assessed by recording leaf chlorophyll fluorescence (Fv/Fm ratio) with a chlorophyll fluorometer OS1-FL (Opti-Sciences, Hudson, NH, USA).

#### 2.2.6. Chlorophyll and Carotenoids

Chlorophyll was extracted from fresh leaves (500 mg) using 80% acetone, and centrifuged at 5702× *g* for 15 min. The supernatant was collected, and the absorbance read at 666, 653, and 470 nm with a spectrophotometer. The pigment concentration was calculated as per the method of Lichtenthaler and Wellburn [25].

### 2.3. Reproductive Traits

#### 2.3.1. Pollen Germination

Pollen grains, collected from flowers of the plants harvested for various treatments, were germinated on a growth medium containing 10% sucrose, 1640 mM boric acid, 990 mM nitrate (pH 6.5), 812 mM magnesium sulfate, and 1269 mM calcium nitrate [22,26]. The percentage germination was recorded.

#### 2.3.2. Pollen Viability

Pollen grains were collected from flowers on the day of anthesis and examined for their viability [27]. The viability of ~200 pollen grains based on their size, shape, and color intensity was assessed in five microscopic fields using 0.5% acetocarmine and expressed as a percentage.

#### 2.3.3. Stigma Receptivity

Stigma receptivity was measured using the esterase test, as per the method of Mattison et al. [28]. Stigmas were harvested from flowers one day prior to anthesis, kept in a solution containing α-NAA (naphthaleneacetic acid) and fast blue B (prepared in phosphate buffer) for 15 min at 37 °C. Stigma receptivity was measured based on color intensity, rated on a 1–5 scale (1-low receptivity, 5-high receptivity).

#### 2.3.4. Ovule Viability

Ovules collected from flowers (one day before anthesis) were tested using a TTC reduction assay for their viability. The ovules were placed on a glass slide, treated with 0.5% TTC prepared in 1% solution, and then transferred to a Petri dish containing two filter papers moistened with distilled water. The ovules were incubated for 15 min at 25 °C in a growth chamber. The resulting red color was rated on a 1–5 scale (1-lowest intensity, 5-highest intensity) [22].

### 2.4. Oxidative Stress and Antioxidants

#### 2.4.1. Malondialdehyde

To measure malondialdehyde (MDA) concentration, fresh tissue (anthers and ovules) was homogenized in 0.1% trichloroacetic acid (TCA) and centrifuged at 3360× *g* for 5 min. The supernatant (0.1 mL) was mixed with 4 mL 0.5% thiobarbituric acid (TBA), prepared in 20% TCA. The mixture was heated at 95 °C for 30 min, cooled in an ice bath, and then centrifuged at 3360× *g* for 10 min at 4 °C. Absorbance of the supernatant was read at 532 nm. MDA concentration was calculated using an extinction coefficient (155 mM cm^−1^) and expressed as nmol g^−1^ DW [29].

#### 2.4.2. Hydrogen Peroxide

Hydrogen peroxide (H_2_O_2_) concentration was measured from fresh tissue (anthers and ovules) extracted in chilled 80% acetone (5 mL), followed by filtration using Whatman filter paper. To this filtrate, 4 mL titanium reagent was added, followed by 5 mL ammonia solution (25%). The mixture was centrifuged at 3360× *g* for 10 min; the residue was dissolved in 1 M H_2_SO_4_. Absorbance of the resulting solution was read at 410 nm. H_2_O_2_ concentration was calculated using an extinction coefficient (0.28 mmol cm^−1^) and expressed as nmol g^−1^ DW [30].

#### 2.4.3. Superoxide Dismutase

Superoxide dismutase (SOD) activity (E.C. 1.15.1.1) was assayed using fresh tissue extracted in a pre-cooled 50 mM phosphate buffer (pH 7.0), which was subsequently centrifuged at 3360× *g* for 5 min at 4 °C. SOD activity was assayed by preparing a reaction mixture comprising 0.1 mL enzyme extract, 50 mM phosphate buffer (pH 7.8), 13 mM methionine, 25 mM nitro blue tetrazolium chloride (NBT), 0.1 mM EDTA (ethylene diamine tetra acetic acid) in 3 mL total volume. Riboflavin (2 mM) was added, and the mixture was kept in fluorescent light (15 W) for 10 min. Absorbance was read at 560 nm, with the activity measured as per [31] and expressed as units mg^−1^ protein.

#### 2.4.4. Catalase

Catalase (CAT) activity (E.C. 1.11.1.6), was assayed by adding 0.1 mL enzyme extract (as above for SOD) to a reaction mixture containing 50 mM phosphate buffer (pH 7.0) and 200 mM H_2_O_2_. Absorbance at 410 nm was recorded for 3 min, with the activity measured using an extinction coefficient (40 mM cm^−1^), expressed as mmol H_2_O_2_ decomposed mg^−1^ protein [32].

#### 2.4.5. Ascorbate Peroxidase

Ascorbate peroxidase (APX) activity (E.C. 1.11.1.11) was assayed by adding 0.1 mL enzyme extract (as above for SOD) to a reaction mixture containing 50 mM phosphate buffer (pH 7), 0.5 mM ascorbic acid, and 0.1 mM EDTA. H_2_O_2_ was added as a substrate. The activity was measured using an extinction coefficient (2.8 mM cm^−1^) [33], expressed as mmol oxidized donor decomposed min^−1^mg^−1^protein.

#### 2.4.6. Glutathione Reductase

Glutathione reductase (GR) activity (E.C. 1.6.4.2) was assayed by adding 0.1 mL enzyme extract (as above for SOD) to a reaction mixture containing 1.5 mL phosphate buffer (100 mM; pH 7.6), 0.2 mL BSA, 0.35 mL NADP (nicotinamide adenine dinucleotide phosphate), and 0.1 mL oxidized glutathione. The enzyme activity was measured as the reduction in absorbance at 340 nm for 3 min, expressed as mmol oxidized donor decomposed min^−1^ mg^−1^ protein [34].

#### 2.4.7. Ascorbic Acid

Ascorbic acid (AsA) concentration was determined using fresh tissue extracted in 6% TCA, followed by centrifugation at 3649.15× *g* for 15 min. To 4 mL supernatant, 2 mL dinitrophenylhydrazine (DNPH; 2%) was added, along with one drop of 10% thiourea. The reaction mixture was boiled in a water bath for 15 min, followed by cooling at room temperature. Pre-cooled H_2_SO_4_ (5 mL) was added, and the absorbance recorded at 530 nm. The AsA concentration was determined from the standard curve and expressed as mg g^−1^ DW [30].

#### 2.4.8. Glutathione

Reduced glutathione (GSH) concentration was assayed from fresh tissue homogenized in 2 mL metaphosphoric acid; the extract was centrifuged at 3650× *g* for 15 min. To 0.9 mL supernatant, 0.6 mL sodium citrate (10%) was added. The assay mixture comprised 100 μL extract, 100 μL distilled water, 100 μL 5,5-dithio-bis-(2)-nitrobenzoic acid (DTNB; 6 mM), and 700 μL NADPH (0.3 mM). To this mixture, 10 μL glutathione reductase (Sigma-Aldrich, Burlington, MO, USA) was added, and the absorbance was read at 412 nm. The GSH concentration was determined from a standard graph and expressed as nmol g^−1^ DW [35].

### 2.5. Soluble Proteins

Plant tissue was oven-dried before extraction with 0.1 M phosphate buffer (pH 7.0) and centrifuged at 514× *g* for 15 min. Protein concentration was measured as per [36] and explained by [37].

### 2.6. Solutes

#### 2.6.1. Proline

Proline concentration was measured in plant tissue using 3% sulphosalicylic acid for extraction, centrifuged at 2150× *g* for 20 min at 4 °C. The supernatant was treated with acidic ninhydrin reagent, and the resulting color read at 520 nm, using toluene as a blank. The concentration was measured as nmol g^−1^ DW [38].

#### 2.6.2. Endogenous γ-Aminobutyric Acid

Endogenous γ-aminobutyric acid (GABA) was measured in fresh tissue homogenized in TCA (8%) and centrifuged at 3360× *g* for 20 min at 25 °C. The supernatant was treated with 4 mL pure diethyl ether, mixed thoroughly for 10 min with a vortexer, followed by centrifugation at 3360× *g* for 20 min. The supernatant was left to sit to evaporate the ether (about 30 min) and tested for GABA concentration, expressed as µmol g^−1^ DW [39].

#### 2.6.3. Trehalose

Trehalose concentration was measured using the method of [40]. The tissue was extracted in 80% hot ethanol, followed by centrifugation at 3360× *g* for 15 min. The supernatant (0.1 mL) was mixed with 2 mL TCA and assayed following the method of [41].

#### 2.6.4. Sucrose

Sucrose concentration was measured in fresh tissue after extraction in 80% ethanol at 80 °C for 1.5 h (twice); the two extracts were combined and evaporated at 40 °C in an air-circulating oven. The sucrose concentration was tested as per [42].

#### 2.6.5. In-Vitro Pollen Germination

Freshly collected pollen grains were tested for germination in a growth medium [37] at 13/7 °C; 12 h/12 h; 24 h) in the presence of 1 mM proline, GABA, sucrose, trehalose, ascorbic acid and reduced glutathione in the growth medium, along with control (not supplemented with any of these molecules).

### 2.7. Statistical Analysis

The experimental design was a 2 factorial randomized block design comprising four contrasting genotypes (two cold-tolerant and two cold-sensitive) and three treatments. There were 15 pots per genotype (two plants per pot) and three replications for each treatment. Five pots in triplicate (15 pots per treatment; 30 plants per treatment) were maintained separately for yield trait measurements. Analysis of variance (ANOVA) for genotype × treatment interactions was performed using Agristat software (Indian Council of Agricultural Research, Goa, India); least significant values (LSD) values were calculated (*p* < 0.05). Tukey’s post hoc test was performed to compare means. In addition, principal component analysis (PCA) was conducted on non-acclimated and acclimated plants to determine the relationships among various measurements.

## 3. Results

### 3.1. Stress Injury to Leaves

#### 3.1.1. Membrane Damage

**Figure 2:**
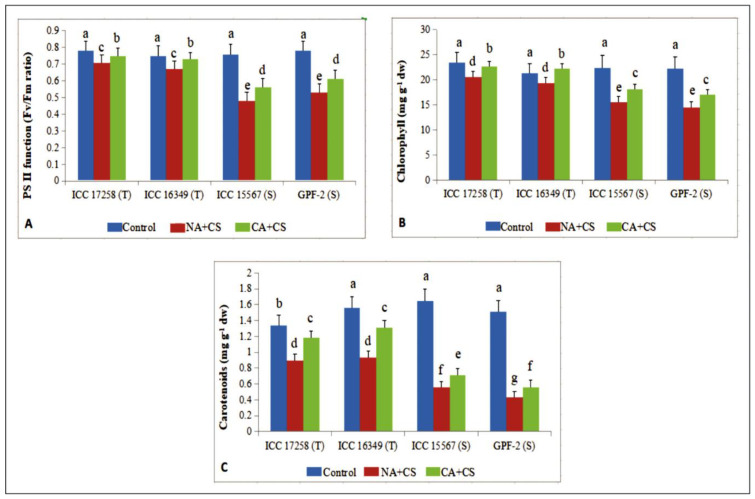
Membrane damage as electrolyte leakage (EL; (**A**)), relative leaf water content (RLWC; (**B**)) and stomatal conductance (*gS*; (**C**)) in leaves of control, non-acclimated, cold stressed; NA + CS) and cold-acclimated, cold stressed (CA + CS) plants of tolerant (T) and sensitive (S) genotypes. Small vertical bars represent standard errors (Mean ± S.E; *n* = 3). Different small letters on vertical bars indicate significant differences from each other (*p* < 0.05; Tukey’s test). Least significant difference (LSD) for interaction (*p* < 0.05): (genotypes × treatments) EL (2.4), RLWC (3.1) and *gS* (18.5).

Cold stress increased membrane damage (as electrolyte leakage; EL) in all four genotypes, more so in cold-sensitive genotypes. Cold-stressed tolerant genotypes had 18.4–20.5% EL (control: 10.5–12.5%), while cold-stressed sensitive genotypes had 26.3–28.3% EL (control: 10.1–13.4%; Figure 2A). Cold acclimation significantly reduced membrane damage in all genotypes, which decreased to 14.5–16.4% in tolerant genotypes and 20.6–21.3% in sensitive genotypes.

#### 3.1.2. Relative Leaf Water Content

Cold stress decreased relative leaf water content (RLWC) to 69.9–70.4% (control: 81.4–82.3%) in cold-sensitive genotypes and 77.5–78.5% in cold-tolerant genotypes (control: 83.4–86.5%; Figure 2B). The RLWC is an indicator of water status of plant. Cold acclimation had a similar effect on RWLC as membrane damage, i.e., the cold-acclimated plants exposed to cold stress significantly improved their RWLC, nearly to the same extent in all genotypes.

#### 3.1.3. Stomatal Conductance

Cold stress did not significantly affect stomatal conductance (*gS*) in non-acclimated cold-tolerant genotypes (Figure 2C), but it decreased in cold-sensitive genotypes (by 13–14%), relative to their respective controls. Cold acclimation significantly increased *gS* in cold-tolerant (8–9%) and cold-sensitive genotypes (17–18%), compared to non-acclimated plants.

#### 3.1.4. Photosystem II Function

**Figure 3:**
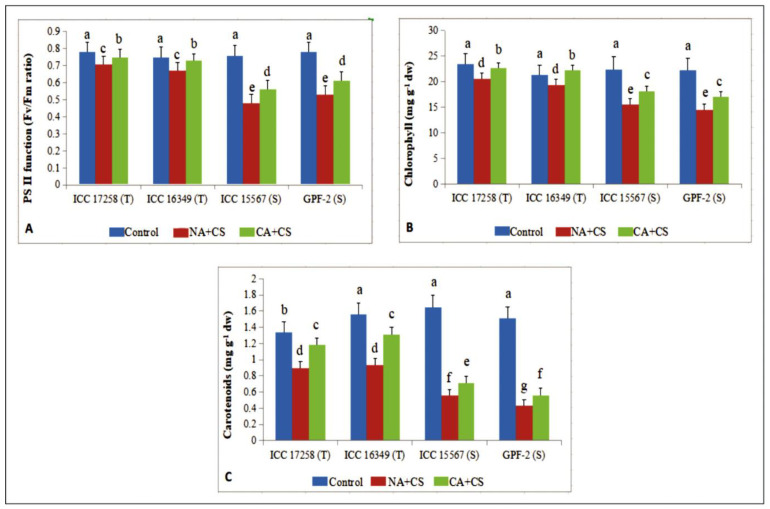
Photosystem II function (PS; (**A**)), chlorophyll (Chl; (**B**)) and carotenoids (Car; (**C**)) in leaves of control, non-acclimated, cold stressed; NA + CS) and cold-acclimated, cold stressed (CA + CS) plants of tolerant (T) and sensitive (S) genotypes. Small vertical bars represent standard errors (Mean ± S.E; *n* = 3). Different small letters on vertical bars indicate significant differences from each other (*p* < 0.05; Tukey’s test). Least significant difference (LSD) for interaction (*p* < 0.05) (genotypes × treatments): PS (0.057), Chl (2.5), Car (0.056).

Photosystem II (PSII) function of control plants ranged from 0.75–0.78 Fv/Fm (variable fluorescence/maximum fluorescence) ratio with no variation between cold-tolerant and cold-sensitive genotypes (Figure 3A). Cold stress decreased PSII function in non-acclimated plants, more so in cold-sensitive genotypes (32–36%) than cold-tolerant genotypes (8–10%) as compared to controls. Cold acclimation significantly enhanced PSII function under cold stress, increasing by 6–8% in cold-tolerant genotypes and 15–16% in cold-sensitive genotypes, relative to non-acclimated plants.

#### 3.1.5. Photosynthetic Pigments

Cold acclimation had a similar effect on chlorophyll (Chl) content as PSII function (Figure 3B). In non-acclimated plants, cold stress significantly decreased Chl in all four genotypes, relative to their respective controls, more so in cold-sensitive genotypes (34–35%) than cold-tolerant genotypes (9–11%). Cold acclimation significantly increased leaf Chl in all four genotypes, relative to non-acclimated plants, more so in cold-sensitive genotypes (16–17%) than cold-tolerant genotypes (10–15%).

Cold had a greater impact on carotenoids than Chl (Figure 3C). In non-acclimated plants, cold stress decreased carotenoid content by 33–40% in cold-tolerant genotypes and 66–71% in cold-sensitive genotypes), compared to their respective controls. Cold acclimation increased carotenoid content in all four genotypes, but unlike PSII function and chlorophyll content, cold-tolerant genotypes increased carotenoids more (32–40%) than cold-sensitive genotypes (26–30%), relative to non-acclimated plants.

### 3.2. Reproductive Traits

**Figure 4:**
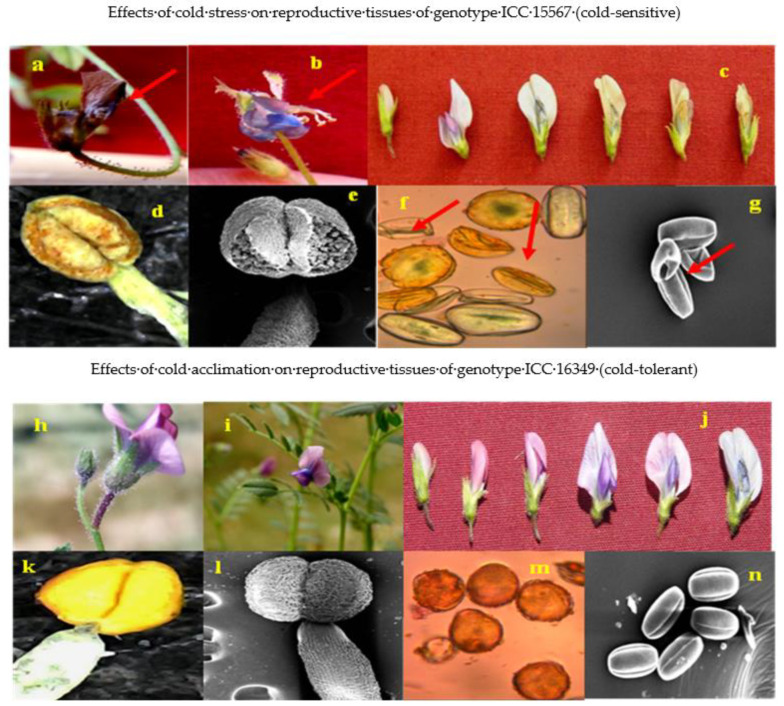
Images showing the effect of cold stress (above) and cold acclimation (below) on chickpea genotypes (reproductive phase). Cold stress effects from genotype ICC 15567 (cold-sensitive): aborted flower (**a**), flower with exposed anthers (**b**), developmental changes leading to abortion of flower (**c**), damaged anthers (**d**,**e**), and distorted and shriveled pollen grains (**f**,**g**). Cold acclimation effects from genotype ICC 16349 (cold-tolerant): flowers (**h**,**i**), developmental changes in cold-acclimated flowers (**j**), cold-acclimated anthers (**k**,**l**), and cold-acclimated viable pollen grains (**m**,**n**).

For reproductive parameters of male or female, cold-tolerant genotypes responded better to cold acclimation than cold-sensitive genotypes. The general effects of cold stress and cold acclimation on flowers, anthers and pollen grains are shown in Figure 4.

#### 3.2.1. Pollen Germination

**Figure 5:**
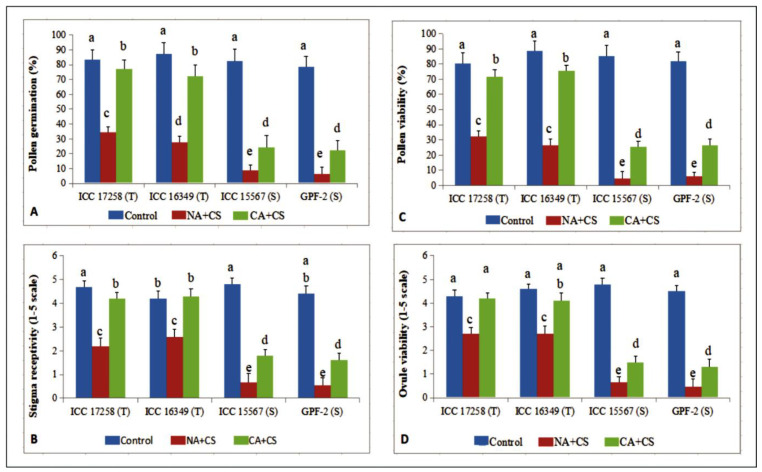
Pollen germination (PG; (**A**)), pollen viability (PV; (**C**)), stigma receptivity (SR; (**B**)) and ovule viability (OV; (**D**)) in control, non-acclimated, cold stressed; NA + CS) and cold-acclimated, cold stressed (CA + CS) plants of tolerant (T) and sensitive (S) genotypes. Small vertical bars represent standard errors (Mean ± S.E; *n* = 3). Different small letters on vertical bars indicate significant differences from each other (*p* < 0.05; Tukey’s test). Least significant difference (LSD) for interaction (*p* < 0.05) (genotypes × treatments): PG (6.9), PV (7.1), SR (0.39), OV (0.36).

Pollen germination in control plants ranged from 78.5–87.5%. Cold stress decreased pollen germination to 27.8–34.6% in non-acclimated cold-tolerant genotypes and 6.4–8.9% in non-acclimated cold-sensitive genotypes (Figure 5A). Cold-acclimated cold-tolerant genotypes had higher pollen germination (72.4–76.9%) than their non-acclimated counterparts, but cold-acclimated cold-sensitive genotypes had poor pollen germination (22.5–25.6%).

#### 3.2.2. Stigma Receptivity

Under cold stress, the female organ in non-acclimated and cold-acclimated chickpea behaved much like the male gametophyte, suggesting that it is also highly sensitive to cold stress. Stigma receptivity was assessed on 1–5 scale using visual scoring. Under cold stress, cold-tolerant genotypes had markedly higher stigma receptivity (2.2–2.6) than cold-sensitive genotypes (1.0) (Figure 5B) but both were markedly lower than the control plants (4.2–4.8). Cold acclimation significantly improved stigma receptivity in all four genotypes, with cold-tolerant genotypes close to control values (4.2–4.3) and cold-sensitive genotypes about one-third of control values (1.6–1.8).

#### 3.2.3. Pollen Viability

Pollen viability in cold-stressed non-acclimated plants decreased to 26.4–32.4% in cold-tolerant genotypes and 4.6–5.9% in cold-sensitive genotypes of their respective controls (81.7–88.9%) (Figure 5C). Cold-acclimated plants exposed to cold stress had much higher pollen viability than cold-stressed non-acclimated plants, being 71.8–75.6% in cold-tolerant genotypes and 25.6–27.3% in cold-sensitive genotypes.

#### 3.2.4. Ovule Viability

Ovule viability was assessed on 1–5 scale using visual scoring. Cold stress significantly reduced ovule viability in all four genotypes, decreasing to 2.2–2.7 in cold-tolerant genotypes and 1.0 in cold-sensitive genotypes, relative to 4.3–4.8 in control plants (Figure 5D). Cold acclimation significantly improved ovule viability under cold stress, more so in cold-tolerant genotypes (4.1–4.2) than cold-sensitive genotypes (1.3–1.5).

#### 3.2.5. Tissue Damage to Anthers and Ovules

**Figure 6:**
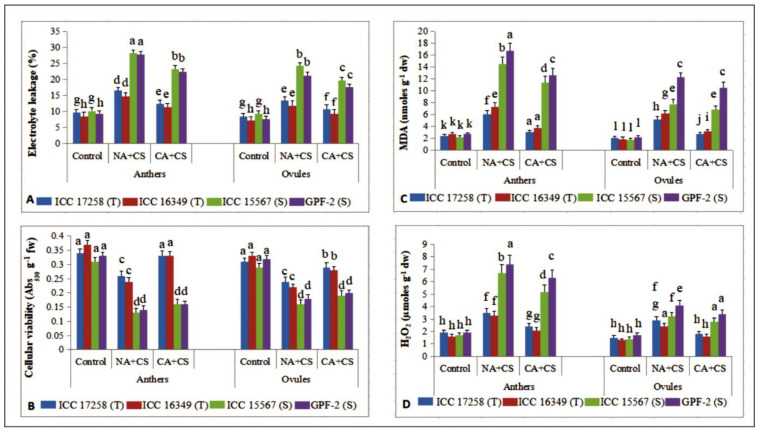
Electrolyte leakage (EL; (**A**)), cellular viability (CV; (**B**)), (Malondialdehyde (MDA; (**C**)) and hydrogen peroxide (H_2_O_2_; (**D**)) concentration in anthers and ovules of control, non-acclimated, cold stressed; NA + CS) and cold-acclimated, cold stressed (CA + CS) plants of tolerant (T) and sensitive (S) genotypes. Small vertical bars represent standard errors (Mean ± S.E; *n* = 3). Different small letters on vertical bars indicate significant differences from each other (*p* < 0.05; Tukey’s test). Least significant difference (LSD) for interaction (*p* < 0.05) (genotypes × treatments): EL (2.4), CV (0.067), MDA (1.8), H_2_O_2_ (0.23).

Cold stress damaged anther and ovule tissues in chickpea, as evidenced from increased electrolyte leakage, expressed as percentage (Figure 6A). Cold-stressed non-acclimated plants of cold-sensitive genotypes had more damage (EL: 27.9–28.3% in anthers, 21.3–24.3% in ovules) than cold-tolerant genotypes (14.7–16.8% in anthers, 11.9–13.6% in ovules), with control plants ranging from 9.3–10.3% in anthers and 7.3–9.2% in ovules. Cold acclimation reduced cold-induced tissue damage in anthers and ovules, more so in cold-tolerant genotypes (11.5–12.4% in anthers, 9.3–10.8% in ovules) than cold-sensitive genotypes (22.4–23.5% in anthers, 17.6–19.8% in ovules).

Cellular viability also decreased with cold stress in anthers and ovules of non-acclimated plants (Figure 6B), more so in cold-sensitive genotypes (56–58% in anthers, 43–45% in ovules over control) than cold-tolerant genotypes (23–35% in anthers, 22–33% in ovules over control). Cold acclimation significantly increased cellular viability to 26–37% in anthers and 21–27% in ovules of cold-tolerant genotypes and 14–23% in anthers and 11–18% in ovules of cold-sensitive genotypes.

### 3.3. Oxidative Stress and Antioxidants

Cold acclimation decreased oxidative stress in anthers and ovules, more so in cold-tolerant genotypes than cold-sensitive genotypes.

#### 3.3.1. Malondialdehyde

Cold stress increased malondialdehyde (MDA) concentration in the anthers and ovules of non-acclimated plants (Figure 6C), while cold acclimation reduced MDA concentration in these tissues. In anthers of cold-stressed non-acclimated plants, MDA concentrations increased more in cold-sensitive genotypes (5.5–7.8-fold) than cold-tolerant genotypes (3.7–4.5-fold), relative to their respective controls. Cold-acclimated plants had significantly lower MDA concentrations in anthers than non-acclimated plants, more so in cold-tolerant genotypes (3–3.5-fold), compared to cold-sensitive genotypes (1.26–1.32-fold).

Control plants had 1.8–2.2 nmoles g^−1^ dw of MDA in ovules, which increased with cold stress, more so in cold-sensitive genotypes (4.3–5.6-fold) than cold-tolerant genotypes (2.4–3.2-fold), over their respective controls. Cold acclimation significantly reduced MDA concentrations in ovules, more so in cold-tolerant genotypes (1.85–1.93-fold) than cold-sensitive genotypes (1.13–1.17-fold), relative to non-acclimated plants.

#### 3.3.2. Hydrogen Peroxide

Control plants had anther H_2_O_2_ concentrations of 1.6–1.9 µmol g^–1^ dw (Figure 6D), increasing under cold stress by 3.8–3.9-fold in cold-sensitive genotypes and 1.84–2.06-fold in cold-tolerant genotypes. Cold acclimation significantly decreased anther H_2_O_2_ concentrations, more so in cold-tolerant genotypes (1.45–1.57-fold) than cold-sensitive genotypes (1.28–1.17-fold), relative to non-acclimated plants. Control plants had ovule H_2_O_2_ concentrations of 1.3–1.7 µmol g^–1^ dw, increasing with cold stress by 1.93–1.84-fold in cold-tolerant genotypes and 2.28–2.41-fold in cold-sensitive genotypes. Cold acclimation decreased H_2_O_2_ concentrations, more so in cold-tolerant genotypes (1.6–1.5-fold) than cold-sensitive genotypes (1.14–1.20-fold), relative to non-acclimated plants.

#### 3.3.3. Superoxide Dismutase

**Figure 7:**
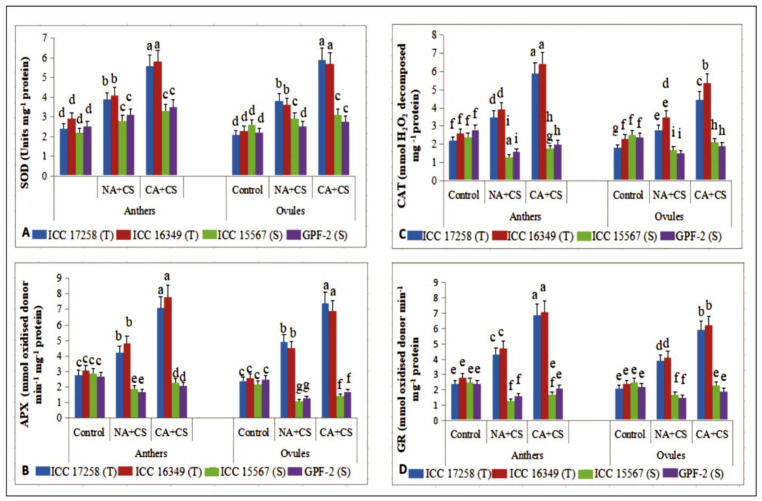
Superoxide dismutase (SOD; (**A**)), ascrobate peroxidase (APX; (**B**)), catalase (CAT; (**C**)) and glutathione reductase (GR; (**D**)) in anthers and ovules of control, non-acclimated, cold stressed; NA + CS) and cold-acclimated, cold stressed (CA + CS) plants of tolerant (T) and sensitive (S) genotypes. Small vertical bars represent standard errors (Mean ± S.E; *n* = 3). Different small letters on vertical bars indicate significant differences from each other (*p* < 0.05; Tukey’s test). Least significant difference (LSD) for interaction (*p* < 0.05) (genotypes × treatments): SOD (0.54), CAT (0.62), APX (0.45), GR (0.49).

Cold stress increased superoxide dismutase (SOD) activity in non-acclimated plants (Figure 7A) by 45–62% in anthers and 56–80% in ovules in cold-tolerant genotypes and 24–27% in anthers and 11–13% in ovules of cold-sensitive genotypes, relative to their respective controls. Cold acclimation increased SOD activity further under cold stress, more so in cold-tolerant genotypes (41–43% in anthers, 55–58% in ovules) than cold-sensitive genotypes (12–17% in anthers, 6–10% in ovules), relative to non-acclimated plants.

#### 3.3.4. Ascorbate Peroxidase

The ascorbate peroxidase (APX) activity in anthers and ovules of control plants ranged from 2.7–3.1 and 2.2–2.6 mmol oxidized donor min^–1^ mg^–1^ protein, respectively (Figure 7B). Cold acclimation had a similar effect on APX activity as CAT activity. Cold-stressed non-acclimated plants increased APX activity in cold-tolerant genotypes (50–54% in anthers, 73–104% in ovules), but decreased APX activity in cold-sensitive genotypes (34–37% in anthers, 48–50% in ovules), relative to the controls. Cold acclimation significantly increased APX activity in all four genotypes, relative to non-acclimated plants, more so in cold-tolerant genotypes (62–69% in anthers, 51–53% in ovules) than cold-sensitive genotypes (21–23% in anthers, 30–31% in ovules).

#### 3.3.5. Catalase

The catalase (CAT) activity in anthers and ovules of control plants ranged from 2.2–2.8 and 1.8–2.4 mmol H_2_O_2_ decomposed mg^–1^ protein, respectively (Figure 7C). Cold stress increased CAT activity by 50–59% in anthers and 52–58% in ovules of cold-tolerant genotypes but decreased CAT activity by 42–45% in anthers and 32–37% in ovules of cold-sensitive genotypes, relative to their respective controls. Cold acclimation increased CAT activity by 64–68% in anthers and 52–58% in ovules of cold-tolerant genotypes and 25–34% in anthers and 23–26% in ovules of cold-sensitive genotypes, relative to non-acclimated plants.

#### 3.3.6. Glutathione Reductase

Cold stress significantly increased glutathione reductase (GR) activity (Figure 7D) in anthers (67–79%) and ovules (67–85%) of non-acclimated plants of cold-tolerant genotypes, but significantly decreased GR activity in cold-sensitive genotypes (33–48% in anthers, 31–33% in ovules), relative to their corresponding controls. Cold acclimation increased GR activity by 51–60% in anthers and 51% in ovules of cold-tolerant genotypes and 30–31% in anthers and 26–35% in ovules of cold-sensitive genotypes, relative to non-acclimated plants.

#### 3.3.7. Ascorbate

**Figure 8:**
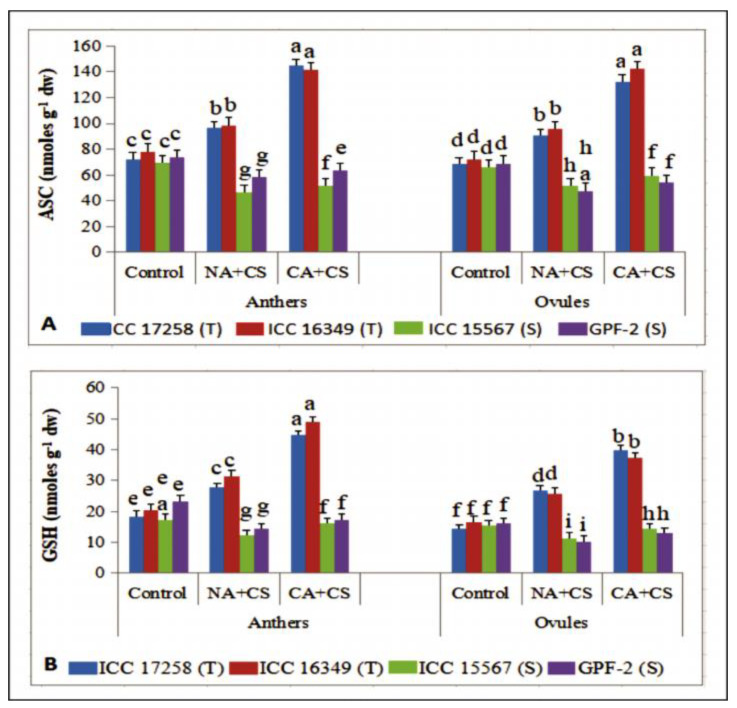
Ascorbate (ASC; (**A**)) and reduced glutathione (GSH; (**B**)) in anthers and ovules of control, non-acclimated, cold stressed; NA + CS) and cold-acclimated, cold stressed (CA + CS) plants of tolerant (T) and sensitive (S) genotypes. Small vertical bars represent standard errors (Mean ± S.E; *n* = 3). Different small letters on vertical bars indicate significant differences from each other (*p* < 0.05; Tukey’s test). Least significant difference (LSD) for interaction (*p* < 0.05) (Genotypes × treatments): ASC (6.9), GSH (3.4).

In non-acclimated plants, cold stress significantly increased ascorbate (ASC) concentration (Figure 8A) in anthers (25–33%) and ovules (31–33%) of cold-tolerant genotypes, but significantly reduced ASC concentration in anthers (20–32%) and ovules (21–31%) of cold-sensitive genotypes, relative to control plants. Cold acclimation significantly improved ASC concentration, more so in anthers (43–50%) and ovules (46–49%) of cold-tolerant genotypes than anthers (8–10%) and ovules (14–15%) of cold-sensitive genotypes, relative to non-acclimated plants.

#### 3.3.8. Glutathione

Cold stress increased glutathione (reduced; GSH) concentrations in anthers (50–53%) and ovules (53–84%) of cold-tolerant genotypes, but decreased GSH concentrations in anthers (28–38%) and ovules (25–36%) of cold-sensitive genotypes, relative to the controls (Figure 8B). Cold acclimation significantly increased GSH levels in anthers and ovules under cold stress, relative to cold-stressed non-acclimated plants, more so in cold-tolerant genotypes (56–60% in anthers, 45–48% in ovules) than cold-sensitive genotypes (20–28% in anthers, 25–27% in ovules).

### 3.4. Cryoprotective Solutes

#### 3.4.1. Proline

**Figure 9:**
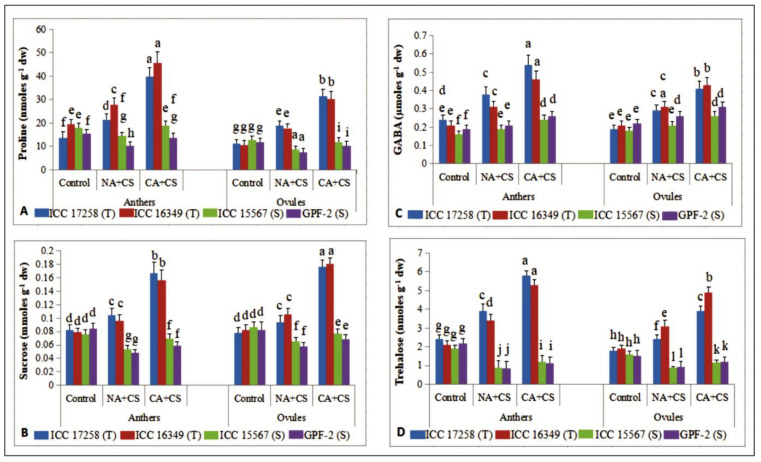
Proline (Pro; (**A**)), Sucrose (Suc; (**B**)), γ-amino butyric acid (GABA; (**C**)) and trehalose (Tre; (**D**)) in anthers and ovules of control, non-acclimated, cold stressed; NA + CS) and cold-acclimated, cold stressed (CA + CS) plants of tolerant (T) and sensitive (S) genotypes. Small vertical bars represent standard errors (Mean ± S.E; *n* = 3). Different small letters on vertical bars indicate significant differences from each other (*p* < 0.05; Tukey’s test). Least significant difference (LSD) for interaction (*p* < 0.05) (genotypes × treatments): Pro (3.9), GABA (0.048), Suc (0.009), Tre (0.40).

Cold stress increased proline (Pro) concentrations in non-acclimated plants of cold-tolerant genotypes (42–57% in anthers, 65–67% in ovules), but decreased proline concentrations in cold-sensitive genotypes (18–32% in anthers, 31–35% in ovules), relative to the controls (Figure 9A). Cold acclimation increased proline concentrations in anthers and ovules, more so in cold-tolerant genotypes (64–85% in anthers, 66–69% in ovules) than cold-sensitive genotypes (30% in anthers, 37–38% in ovules), relative to non-acclimated plants.

#### 3.4.2. Sucrose

Cold stress increased sucrose concentrations by 21–26% in anthers and 20–28% in ovules of cold-tolerant genotypes, but decreased sucrose concentrations by 28–42% in anthers and 24–29% in ovules of cold-sensitive genotypes, relative to their respective controls (Figure 9B). Cold acclimation increased sucrose concentrations more in cold-tolerant genotypes (60–62% in anthers, 71–87% in ovules) than cold-sensitive genotypes (23–27% in anthers, 17–18% in ovules), relative to non-acclimated plants.

#### 3.4.3. γ-. Aminobutyric Acid

Cold stress increased γ-aminobutyric acid (GABA) concentrations more in cold-tolerant genotypes (47–58% in anthers, 46–52% in ovules) than cold-sensitive genotypes (10–18% in anthers, 16–18% in ovules), relative to their respective controls (Figure 9C). Cold acclimation further increased GABA concentrations in all four genotypes, more so in cold-tolerant genotypes (42–48% in anthers, 39–41% in ovules) than cold-sensitive genotypes (23–26% in anthers, 19–23% in ovules), relative to non-acclimated plants.

#### 3.4.4. Trehalose

Cold stress significantly increased trehalose levels in anthers (61–62%) and ovules (33–63%) of cold-tolerant genotypes, but significantly reduced trehalose levels in anthers (53–60%) and ovules (39–45%) of cold-sensitive genotypes, relative to the controls (Figure 9D). Cold acclimation increased trehalose concentrations in all four genotypes, more so in cold-tolerant genotypes (48–55% in anthers, 58–62% in ovules) than cold-sensitive genotypes (31–35% in anthers, 31–33% in ovules), compared to non-acclimated plants.

### 3.5. Yield Traits

**Figure 10:**
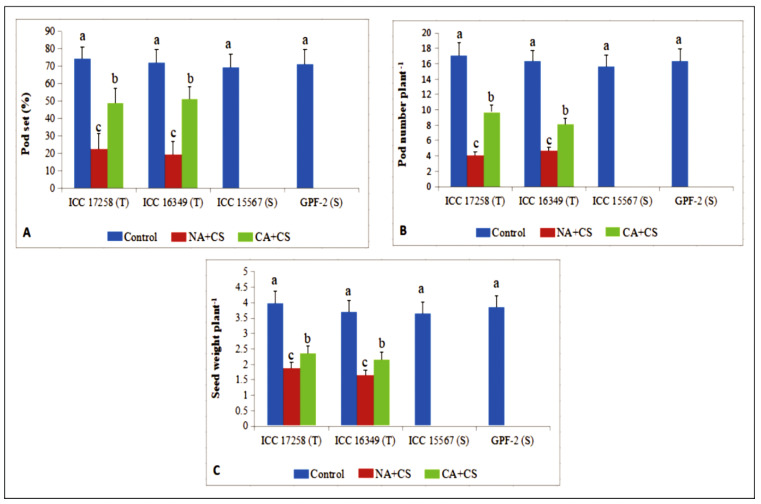
Pod set (**A**), pod number plant^−1^ (**B**) and seed weight plant^−1^ (**C**) in plants of control, non-acclimated, cold stressed; NA + CS) and cold-acclimated, cold stressed (CA + CS) plants of tolerant (T) and sensitive (S) genotypes. Small vertical bars represent standard errors (Mean ± S.E; *n* = 3). Different small letters on vertical bars indicate significant differences from each other (*p* < 0.05; Tukey’s test). Least significant difference (LSD) for interaction (*p* < 0.05) (Genotypes × treatments): Pod set (10.3), Pod number (1.8), Seed weight (0.41).

Pod set in control plants was 72.1–74.5% in cold-tolerant genotypes and 69.4–71.4% in cold-sensitive genotypes (Figure 10A). Cold stress decreased pod set to 19–22% in cold-tolerant genotypes and zero in cold-sensitive genotypes. Cold acclimation increased pod set under cold stress to 48.9–51.4% in cold-tolerant genotypes but had no effect on pod set in cold-sensitive genotypes.

Control plants of cold-tolerant and cold-sensitive genotypes produced 16.3–17.1 and 15.6–16.3 pods per plant, respectively (Figure 10B). Cold stress decreased pod number by 70–76% (4.1–4.7 pods plant^–1^) in cold-tolerant genotypes, relative to the control plants, but cold-sensitive genotypes did not produce any pods. Cold acclimation improved pod set under cold stress by 70–76% (8.1–9.7 pods per plant) in cold-tolerant genotypes, relative to non-acclimated plants, while cold-sensitive genotypes did not produce any pods.

Control plants of cold-tolerant genotypes yielded 3.65–3.98 g plant^–1^, which decreased by 52–55% under cold stress (no yield in cold-sensitive genotypes) (Figure 10C). Cold acclimation increased seed yield in cold-tolerant genotypes under cold stress, without any effect on cold-sensitive genotypes. Cold acclimation improved seed yield by 25–31% (to 2.16–2.35 g plant^–1^) in cold-tolerant genotypes under cold stress, relative to non-acclimated plants.

### 3.6. Effect of Cryoprotective Solutes and Antioxidants on In-Vitro Pollen Germination

Pollen germination in control plants of cold-tolerant genotypes was 85.6–88.15% and cold-sensitive genotypes was 79.6–82.1%. Cold stress decreased pollen germination to 31.3–35.6% in cold-tolerant genotypes and 6.8–8.9% in cold-sensitive genotypes (Table 1). Exogenous supplementation of ascorbate, GSH, proline, trehalose, and sucrose improved pollen germination markedly in all four genotypes, more so with sucrose supplementation, followed by GABA and ascorbate.

### 3.7. Principal Component Analysis

#### 3.7.1. Non-Acclimated (NA) Plants

**Figure 11:**
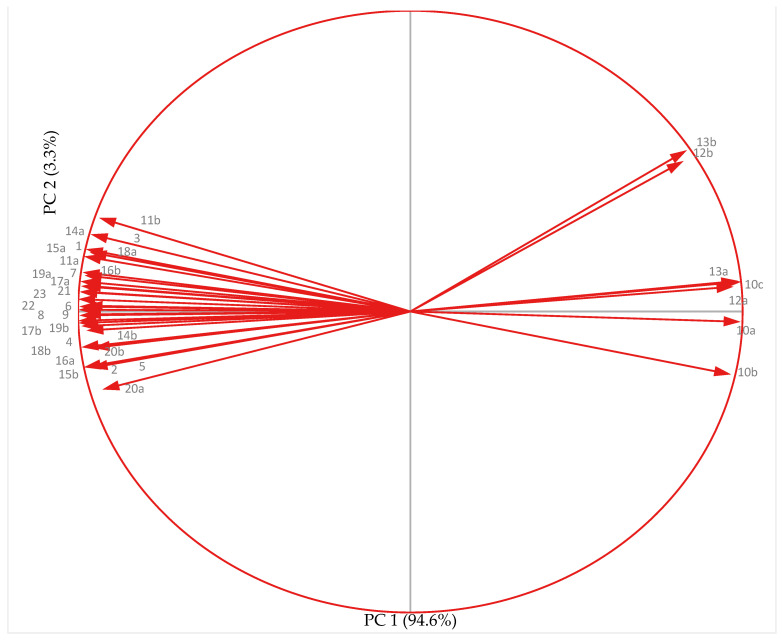
Principal component analysis of yield, reproductive, biochemical and leaf traits in chickpea genotypes under non acclimated conditions. Abbreviations: a-anther, b-ovule; 1-RLWC-relative leaf water content; 2-SC-stomatal conductance; 3-PSII-photosynthetic efficiency; 4-Chl-chlorophyll content; 5-CAR-carotenoids; 6-PG-pollen germination; 7-PV-pollen viability; 8-SR-stigma receptivity; 9-OV-ovule viability; 10-EL-electrolyte leakage; 11-CV-cellular viability; 12-MDA-malondialdehyde; 13-H_2_O_2_-hydrogen peroxide; 14-SOD-superoxide dismutase; 15-CAT- catalase; 16-APX-ascorbate peroxidase; 17-GR-glutathione reductase; 18-ASC-ascorbic acid; 19-GSH-glutathione; 20-proline; 21-pod set %; 22- pod number plant^–1^; 23-seed weight plant^–1^.

Principal component analysis (PCA; Figure 11) graph for the chickpea genotypes grown under non-acclimated temperature conditions revealed a significant positive relationship among yield traits (pod set %, pod number plant^−1^ and seed weight and seed number plant^−1^), reproductive traits (pollen germination, PG; pollen viability, PV; stigma receptivity, SR; ovule viability, OV; cellular viability, CV), leaf traits (stomatal conductance, *gS*; relative leaf water content, RLWC; chlorophyll, Chl; carotenoids, CAR; chlorophyll fluorescence, PSII) and biochemical traits (SOD, CAT, APX, GR, ASC, GSH and proline). All these traits were found to strongly correlate with each other except electrolyte leakage (EL) MDA, H_2_O_2_ that indicated negative correlation with cold tolerance.

PCA revealed that PC1 and PC2 accounted for 97.9% of the variation (PC1: 94.6% and PC2: 3.3%). PC1 showed EL in leaves, anthers and ovules, MDA and H_2_O_2_ in anthers and ovules. PC2 showed yield traits (pod set %, pod number plant^−1^, seed weight and seed number plant^−1^), reproductive traits (PG, PV, SR and OV), stress injury traits (*gS*, RLWC, CV, Chl, CAR, and PSII) and biochemical traits (SOD, CAT, APX, GR, ASC, GSH and proline).

The indices here formed three groups; Group 1 had six indices: proline (anthers and ovules), GR (ovules), ASC (ovules), APX (anthers), chlorophyll content (Chl, leaves), CAT (ovules and anthers). Group-2 included 13 indices: CV (anthers and ovules), PSII (leaves), ASC (anthers), SOD (anthers), RLWC, PV, PG, APX (anthers and ovules), GR (anthers and ovules), GSH (ovules), OV, pod number per plant and seed weight per plant. A strong and positive correlation was noticed in Group 1 and Group 2 with an acute angle, thus, suggesting that any of these traits may probably be used to measure the association of various traits with yield plant^–1^. Group 3 consisted of H_2_O_2_, MDA (anthers and ovules), and EL (leaves, anthers and ovule), which had a negative association with yield per plant as well as with indices in Groups 1 and 2.

Narrow vector angles in the PC1-dominating variables in the arc from H_2_O_2_ (ovules) to EL (ovules) reveal strong correlations between these variables (H_2_O_2_, MDA (anthers and ovules) and EL (leaves, anther and ovule)). These traits indicate the low temperature injury to membranes and oxidative damage to the chickpea genotypes and are negatively correlated with other traits (yield and biochemical traits). The traits such as RLWC, Chl, PSII, CV, proline (anthers and ovules), SOD (anthers and ovules), CAT (ovules and anthers), APX (anthers and ovules), GR (anthers and ovules), ASC (anthers and ovules), GSH (anthers and ovules) were strongly correlated with yield traits (pod number plant^–1^ and seed weight plant^–1^) and reproductive traits (PV, PG, SR, and OV). Hence, it can be concluded that these traits of leaves, anthers ovules and pollen grains would be useful as indicators of yield under non-acclimated cold stress conditions in chickpea.

#### 3.7.2. Cold-Acclimated Plants

**Figure 12:**
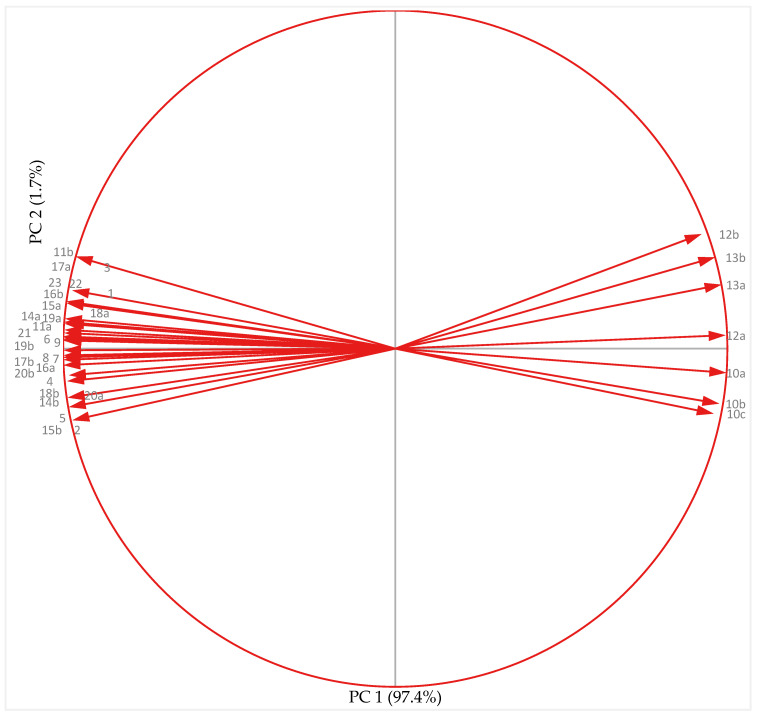
Principal component analysis of yield, reproductive, biochemical and leaf traits in chickpea genotypes under cold-acclimated conditions. a-anther, b-ovule; 1-RLWC-relative leaf water content; 2-SC-stomatal conductance; 3-PSII-photosynthetic efficiency; 4-Chl-chlorophyll content; 5-CAR-carotenoids; 6-PG-pollen germination; 7-PV-pollen viability; 8-SR-stigma receptivity; 9-OV-ovule viability; 10-EL-electrolyte leakage; 11-CV-cellular viability; 12-MDA-malondialdehyde; 13-H_2_O_2_-hydrogen peroxide; 14-SOD-superoxide dismutase; 15-CAT-catalsae; 16-APX-ascorbate peroxidase; 17-GR-glutathione reductase; 18-ASC-ascorbic acid; 19-GSH-glutathione; 20-proline; 21-pod set %; 22-pod number plant^–1^; 23-seed weight plant^–1^.

Principal component analysis (PCA; Figure 12) for the chickpea genotypes grown under cold-acclimated conditions revealed a significant positive relationship among yield traits (pod set %, pod number plant^–1^ and seed weight plant^–1^), reproductive traits (PG, PV, SR, OV, CV), leaf traits (*gS*, RLWC, Chl, CAR, and PSII) and biochemical traits (SOD, CAT, APX, GR, ASC, GSH and proline). All these traits showed strong correlation to each other except MDA, H_2_O_2_, and EL that indicated association of these traits with low temperature damage to vegetative and reproductive tissues.

PCA showed that PC1 and PC2 accounted for 99.2% of the variation (PC1: 97.8% and PC2: 1.4%). PC1 described EL (leaves, anthers and ovules), MDA and H_2_O_2_ (anthers and ovules). Yield traits (pod set %, pod number plant^–1^ and seed weight plant^–1^), reproductive traits (PG, PV, SR, OV), stress injury traits (*gS*, RLWC, CV, Chl, and PSII) and biochemical traits (SOD, CAT, APX, GR, ASC, GSH, H_2_O_2_ (ovules) and proline (anthers and ovules)) as indicated on PC2. The indices here formed three groups with Group-1 comprising of six indices: proline (anthers and ovules), GR, ASC (ovules), APX (anthers), Chl, CAT (ovules and anthers). Group-2 included 13 indices: CV (anthers and ovules), PSII, ASC (anthers), SOD (anthers), RLWC, PV, PG, APX (anthers and ovules), GR (anthers and ovules), GSH (anthers and ovules), OV, pod number plant^–1^ and seed weight plant^–1^. The indices in Group-1 and Group-2 were strongly and positively correlated with an acute angle and indicated that any of these indices can probably be used to correlate antioxidative activity and yield plant^–1^. There was third group of H_2_O_2_, MDA (anthers and ovules), and EL (leaves, anthers and ovule), which represent a weak link with indices in Group 1 and 2.

Narrow vector angles in the PC1-dominating variables, described in the arc from MDA (ovule) to EL (leaf) reveal strong correlations between H_2_O_2_, MDA (anthers and ovules) and EL (leaves, anthers and ovules). These traits indicate low temperature injury to chickpea genotypes, therefore are negatively correlated with other traits (yield and biochemical traits). Since, proline (anthers and ovules), GR (anthers and ovules), ASC (anthers and ovules), APX (anthers and ovules), chlorophyll (Chl), CAT (ovules and anthers), CV (anthers and ovules), PSII, SOD (anthers and ovules), RLWC, GR (anthers and ovule), GSH (anthers and ovules) were strongly correlated with yield traits (pod number plant^–1^ and seed weight plant^–1^) and reproductive traits (PV, PG, SR, OV), it can be concluded that these traits of leaves, anthers, ovules and pollen grains would be useful as indicators of yield under cold-acclimated conditions.

## 4. Discussion

As a winter season crop in several parts of the world, chickpea suffers from cold-stress-induced damage to vegetative and reproductive tissues. Studies have reported beneficial effects of cold acclimation for chickpea during the seedling or early vegetative phase [9,21,43], there are no reports investigating the impact of cold acclimation during the reproductive stage. In the present study, cold acclimation improved leaf, anther, and ovule function under cold stress, relative to non-acclimated plants, suggesting that cold acclimation is advantageous for vegetative and reproductive organs, improving plant growth, reproduction, and yield. Our study also showed that cold acclimation improved the response of vegetative tissues (leaves) to cold stress by reducing cold-induced damage and improving cellular function, such as membrane damage or relative leaf water content, stomatal conductance, PSII function, or leaf chlorophyll and carotenoid concentrations.

Cold acclimation can improve hardiness to cold stress [17] through various mechanisms. Cold acclimation can reduce membrane damage by increasing the ratio of unsaturated to saturated fatty acids, as reported in 20-day old chickpea seedlings [20]. We observed improved leaf water status in cold-acclimated chickpea plants, as reported in barley [18], and could be due to better root hydraulic conductivity and osmolyte accumulation [44]. The observed reduction in chlorophyll loss of cold-acclimated chickpea plants might have resulted from augmented leaf water status and reduced oxidative damage [9]. The reduction in chlorophyll and PSII function agrees with previous studies on cold-acclimated chickpea seedlings [43] and *Arabidopsis thaliana* (accession C24) [19] exposed to cold stress. Carotenoids are vital for maintaining the leaf redox status, protecting them from photoinhibition under cold stress [45], in our study, cold acclimation increased leaf carotenoid concentrations in cold-stressed chickpea, which might have protected the leaves from photoinhibition by adjusting the redox status and keeping the leaves photosynthetically active.

In non-acclimated chickpea plants exposed to cold stress, the marked reductions in growth, pod set, yield-related traits (pod and seed weights), and reproductive function could be associated with increased membrane damage and decreased water status, stomatal conductance, chlorophyll concentration, and PSII function in leaves. In chickpea, low temperature stress increased membrane damage [5] and decreased leaf hydration status and stomatal conductance could be due to reduced root hydraulic conductivity [46,47], chlorophyll [48], chlorophyll fluorescence [43], pollen function, stigmatic and ovular activity [4,8,49], and pod set and yield traits [7,10] Cold-stress-induced membrane disruption results from altered lipid–protein interactions [50] or lipid peroxidation [51], chlorophyll loss in cold-stressed plants, as observed in our study, might be due to inhibited chlorophyll synthesis or increased chlorophyll degradation [52] or photooxidation-induced disorganization of chloroplasts [53], which consequently decreases chlorophyll fluorescence [43]. Leaf damage due to cold stress can disrupt photosynthetic function and sucrose synthesis and transport to developing floral organs, causing impaired reproductive function and reduced yields [54].

Unlike leaf tissues, the response of reproductive organs to cold acclimation in cold-tolerant and cold-sensitive chickpea genotypes differed. The zero pod set and zero yield in cold-acclimated cold-sensitive genotypes under cold stress indicates the lack of a cold acclimatization response. In contrast, cold-tolerant genotypes had a cold acclimation response (increased pod and seed set relative to non-acclimated plants). Interestingly, our findings and those of [9] indicate that vegetative and reproductive tissues of cold-sensitive chickpea genotypes differ in their response to cold acclimation. Indeed, reproductive organs (anthers and ovules) had significantly more tissue damage and less cellular viability in cold-sensitive genotypes than cold-tolerant genotypes; moreover, these organs were less responsive to cold acclimation in sensitive genotypes. Thus, the differential response to cold acclimation might lie in the tissue sensitivity of floral organs in cold-sensitive and cold-tolerant genotypes, as indicated by various traits related to tissue damage, but this aspect needs further study.

In chickpea, cold stress reduces pollen viability, pollen load on stigma, stigma receptivity, and ovule viability [4,49]. Chickpea plants fail to set pods at temperatures <20/10 °C due to various abnormalities related to developmental and functional factors [1,4,8,49]. In cold-tolerant chickpea genotypes, cold acclimation reduced the adverse effect of cold stress, increasing yield. Little or no cold acclimation of reproductive organs in cold-sensitive genotypes might be due to poor expression of enzymatic and non-enzymatic antioxidants and reduced accumulation of cryoprotective molecules in reproductive organs. The cold-sensitive genotypes were unable to significantly reduce cold-stress-induced oxidative stress markers, such as MDA and H_2_O_2_, in both male and female reproductive organs following acclimation. Consequently, these genotypes failed to detoxify ROS following the production of those by lower temperatures, impairing male and female gamete function and causing flower/pod abortion.

The role of ROS is well documented for sensitivity to abiotic stresses [55]. In chickpea, cold stress affects male and female gamete function, resulting in poor pollen germination, viability, stigmatic receptivity, and ovule viability [4,9,49,56]. The current study showed that cold stress caused tissue damage in anthers and ovules and reduced their cellular viability. The manifold increase in oxidative stress in anthers and ovules under cold stress points to its role in tissue damage and cell viability in these organs. Therefore, it cannot be ruled out that oxidative-stress-induced tissue damage disrupts developmental and functional aspects of anthers and ovules. In rice anthers, ROS accumulation has been reported under drought [57], and heat stress [58]. In cytoplasmic male sterile (CMS) rice material, the CMS line (sterile anthers) had significantly higher ROS concentrations in anthers than the corresponding maintainer line (fertile anthers) [59,60].

The cold acclimation response of cold-tolerant chickpea genotypes could be attributed to a substantial reduction in MDA and H_2_O_2_levels in anthers and ovules and increased accumulation of antioxidants (enzymatic and non-enzymatic). An increase in enzymatic and non-enzymatic antioxidant levels reduced the oxidative species generated under cold stress, thus reducing the oxidative stress in anthers and ovules to levels too low to cause considerable damage to these organs. Thus, reduced oxidative damage to these organs improved anther and ovule performance under cold stress in cold-acclimated plants, relative to non-acclimated plants in the cold-tolerant genotypes. Decreased production of oxidative species and increased production of antioxidants leads to cold tolerance in crops such as rice (*Oryza sativa* L.) [61] and *Brassica* sp. [62]. Cold acclimation improved the antioxidant capacity of barley [63] and chickpea [64] leaves.

Numerous studies have demonstrated that the antioxidant enzyme system in plants can protect against ROS, but little is known about antioxidant enzymes in developing anthers [4], or the interaction between cold-induced ROS concentrations in anthers and ovules of chickpea. In some crops, antioxidant enzymes reduce ROS-induced damage and are important components of plant tolerance to environmental stresses [65,66]. In the present study, the activities of SOD (causes dismutation of peroxides), CAT (detoxifies the hydrogen peroxide), APX (detoxifies hydrogen peroxide using ascorbate as a substrate), and GR (catalyzes the reduction of glutathione disulfide to the sulfhydryl form GSH) increased in anthers and ovules of non-acclimated cold-tolerant genotypes, indicating an inherent ability of these genotypes to reduce cold-induced oxidative stress. However, the reduction in pod numbers in cold-tolerant non-acclimated genotypes exposed to cold stress suggests that the decrease in oxidative damage in anthers and ovules was not significant. In contrast, cold-sensitive genotypes had much lower antioxidant levels in anthers and ovules than cold-tolerant genotypes, causing severe oxidative damage to these organs, manifested as inhibited reproductive function and lack of pod set. The considerably greater reduction in tissue damage (as EL and cellular viability) in anthers and ovules of cold-tolerant genotypes than cold-sensitive genotypes might be due to an improvement in unsaturation of lipids [67], and reduction in oxidative stress in acclimated plants. Like anthers and ovules, cold acclimation reduced the severity of oxidative stress in chickpea seedlings [21] and barley leaves [63]. Variations, however, have been reported in the activities and the type of antioxidants in cold-acclimated plants, which might depend on the experimental conditions and plant species used [63,68]. In the present study, components of the ascorbate–glutathione pathway were greatly expressed, compared to other antioxidative enzymes, suggesting their larger role in the cold acclimation potential of cold-tolerant genotypes.

Cryoprotective molecules can maintain reproductive function in plants. Following cold acclimation, the anthers and ovules of cold-sensitive genotypes accumulated lesser amounts of cryoprotective molecules, such as proline, GABA, trehalose, and sucrose, compared to cold-tolerant genotypes. Our previous study [8] on cold-stressed chickpea revealed an association between reduced carbohydrates in ovules and floral abortion. The cold acclimation of cold-tolerant genotypes can thus be attributed to the inherent ability of these genotypes to reduce oxidative stress and enhance antioxidant levels (enzymatic and non-enzymatic) and cryoprotective solutes in reproductive organs (anthers and ovules), improving reproductive function, e.g., pollen viability, pollen load on stigma, stigma receptivity and ovule viability, and subsequently number of pods and seeds.

Cold acclimation can enhance endogenous proline (*Chrysanthemum* sp.) [69], carbohydrates (safflower, *Carthamus tincotorius*) [70], and GABA (barley and wheat; [71] levels in plants. Cryoprotective solutes, such as amino acids (proline, GABA) and carbohydrates (sucrose, trehalose), play diverse roles in plant cells [72]. Moreover, its role as an osmolyte in osmotic adjustment, proline stabilizes membranes and proteins, scavenges free radicals, and buffers cellular redox potential under stress conditions [73]. The importance of proline in cold stress mitigation can be judged because it has been used as a biomarker of cold tolerance [74]. In cold-tolerant chickpea under cold stress, higher proline levels were attributed to increased expression of the gene responsible for proline transport, *proline transporter 1* [4]. GABA is a non-protein amino acid—it has a signaling role with functions to protect from oxidative stress, maintain C and N mechanism, regulate pH in cytosol, and in osmoregulation [75] and cold tolerance [76]. Trehalose (α-D-glucopyranosyl- α-D-glucopyranoside) is a vital compatible sugar solute—it has a signaling role and stabilizes lipid membranes, dehydrated enzymes, and proteins during desiccation [77]. It has also been implicated in acquiring stress tolerance in plants, including cold stress [78]. Sucrose has been implicated in conferring cold tolerance [79] and can directly protect cell membranes by interacting with the phosphate in their lipid headgroups, thus decreasing membrane permeability [80]. Non-acclimated cold-tolerant chickpea genotypes had substantially higher levels of these solutes than non-acclimated cold-sensitive genotypes, suggesting their involvement in cold tolerance. However, their concentrations may have been inadequate to maintain reproductive competence. The depletion of proline, sucrose, and reducing sugars in flowers due to impaired mobilization and synthesis causes flower abortion due to decreased pollen viability and retarded pollen tube growth [9,56].

Sucrose, in addition to a cryoprotectant, might act as a source of carbon to developing anthers and ovules. Adequate carbohydrate supply is critical for anther function under cold stress [81] and sucrose is an important carbohydrate molecule required for proper anther function, especially under stress, e.g., in tomato (*Solanum lycopersicum*) [82] and chickpea [8]. In an earlier study, the expression of sucrose-synthesizing genes was compared in anthers of cold-stressed cold-tolerant and cold-sensitive chickpea genotypes [4]. Under cold stress, the anthers of cold-tolerant genotype, ICC 16349, had higher pollen viability than cold-sensitive, GPF2. Increased pollen viability in the cold-tolerant genotype was associated with up-regulation of sucrose-synthesizing genes, *UDP glucose pyrophosphorylase*, *sucrose phosphate synthase2,* and *CWIN cell wall invertase* [4].

PCA graphs of non-acclimated and cold-acclimated treatments of chickpea genotypes demonstrated strong correlation among reproductive, biochemical, anti-oxidative and yield traits. At the same time, cold-acclimated plants showed increased protective traits (CAR, Chl, CV, SC, PSII, CAT, SOD, APX, GR, ASC, proline) as compared to non-acclimated plants so that plants could achieve cold tolerance. Furthermore, cold-acclimated plants showed higher reproductive traits (PG, PV, SR, OV) than non-acclimated plants that may result in enhanced yield traits (pod set %, pod number plant^–1^, seed weight plant^–1^). In contrast, non-acclimated plants showed significant chilling injury traits (EL, MDA, H_2_O_2_) as compared to cold-acclimated plants. Moreover, there was a strongly positive correlation among various protective, reproductive and yield traits in cold-acclimated plants as compared to non-acclimated plants. Thus, cold-acclimated plants acquired substantial cold tolerance that leads to increased yield.

## 5. Conclusions

Vegetative and reproductive tissues respond to cold acclimation in chickpea. However, the degree of responsiveness varies between the tissues in cold-tolerant and cold-sensitive genotypes. Following cold acclimation, the leaves (vegetative) of cold-tolerant and cold-sensitive genotypes had less cold-induced membrane damage and improved cellular function (relative leaf water content, stomatal conductance, PSII function, chlorophyll and carotenoid contents) under cold stress. The degree of responsiveness of reproductive organs (anthers and ovules) to cold acclimation in cold-tolerant and cold-sensitive chickpea genotypes varied, with little to no response of cold-sensitive genotypes (zero pod set and zero yield under cold stress), while cold-tolerant genotypes improved pod set and seed yield, relative to non-acclimated plants. In cold-sensitive genotypes, the lack of cold acclimation resulted from the inability of anthers and ovules to reduce oxidative stress either through the reduced generation of oxidative molecules or enhanced production of enzymatic and non-enzymatic antioxidants in both reproductive tissues. The anthers and ovules of cold-sensitive genotypes also failed to produce enough cryoprotective solutes (proline, GABA, trehalose, and sucrose), instrumental in reducing cold-induced damage, and thus had more tissue damage, less cellular viability and lower pollen and ovule viability, pollen load on stigma, and stigma receptivity than cold-tolerant genotypes. In contrast, the responsiveness of cold-tolerant genotypes to cold acclimation resulted from their ability to produce lower amounts of oxidative molecules and increased activity/amounts of antioxidants and cryoprotective solutes in anthers and ovules, reducing damage to anthers and ovules to maintain their viability and reproductive function under cold stress, leading to improved pod set and seed yield, relative to non-acclimated plants. PCA analysis of the non-acclimated and cold-acclimated conditions cold-stressed chickpea plants revealed similarity in types of various antioxidants and cryo-protective solutes required in imparting a stable reproductive function to confer cold tolerance. However, the expression of these molecules was much stronger in cold-acclimated plants, which minimized the oxidative damage. We conclude that cold tolerance in chickpea appears to be related to the better ability of anthers and ovules to acclimate to cold stress through various antioxidants and cryoprotective solutes. This information will be useful in developing genetic, molecular, breeding and agronomic management practices toward increasing cold tolerance in chickpea.

## Figures and Tables

**Table 1:** Effect of various solutes and non-enzymatic antioxidants on pollen germination under cold stress in tolerant (T) and sensitive (S) chickpea genotypes.

Treatment	ICC 17258 (T)	ICC 16348 (T)	ICC 15567 (S)	GPF2 (S)
Control	85.6 ± 5.9 a	88.1 ± 5.1 a	82.1 ± 4.8 a	79.6 ± 5.5 a
Cold-stressed (CS) (13/7 °C;12 h/12 h; 1 d)	35.6 ± 4.9 d	31.3 ± 3.6 d	8.9 ± 2.1 f	6.8 ±1.6 f
CS + Proline (1mM)	53.5 ± 4.8 cd	52.4 ± 5.4 d	21.9 ± 3.4 ef	23.4 ± 2.5 ef
CS + GABA (1 mM)	61.3 ± 4.3 bc	60.1 ± 4.7 bc	33.5 ± 2.3 e	31.5 ± 2.1 e
CS + Sucrose (1 mM)	65.6 ± 4.2 b	62.4 ± 4.6 b	31.3 ± 2.1 e	34.2 ± 3.2 e
CS + Trehalose (1 mM)	54.5 ± 3.5 d	52.4 ± 4.4 d	29.5 ± 3.1 e	31.2 ± 3.3 e
CS + Ascorbate (1 mM)	53.8 ± 3.2 d	56.1 ± 3.7 cd	28.7 ± 2.7 e	31.3 ± 2.9 e
CS + Reduced Glutathione (1 mM)	56.4 ± 3.5 cd	53.2 ± 2.9 d	26.3 ± 2.4 ef	29.6 ± 2.2 e

Different small letters indicate significant differences from each other (*p* < 0.05; Tukey’s test).

## Data Availability

The data presented in this study are available in article.
